# Healthcare Workers' Resilience Mediates the Influence of Organizational Commitment and Anxiety Response to Viral Epidemic on Their Quality of Life in the COVID-19 Pandemic

**DOI:** 10.3389/fpsyt.2021.735016

**Published:** 2022-01-04

**Authors:** Hoon Sung Son, Kyumin Kim, Inn-Kyu Cho, Joohee Lee, Jung Mun Choi, Kwang Hyun Kil, Jiyeon Kim, Youjin Hong, Myung Hee Ahn, Seockhoon Chung

**Affiliations:** ^1^Department of Psychiatry, Asan Medical Center, University of Ulsan College of Medicine, Seoul, South Korea; ^2^Department of Arts in Literature and Art Therapy, Graduate School of Konkuk University, Seoul, South Korea; ^3^Asan Academic Institute, Asan Medical Center, Seoul, South Korea; ^4^Department of Art Therapy, Hanyang Cyber University, Seoul, South Korea; ^5^Department of Psychiatry, Gangneung Asan Hospital, University of Ulsan College of Medicine, Gangneung, South Korea; ^6^Division of Psychiatry, Health Screening and Promotion Center, Asan Medical Center, Seoul, South Korea

**Keywords:** health personnel, psychological, COVID-19, work engagement, quality of life

## Abstract

**Objective:** In the COVID-19 pandemic era, healthcare workers suffer from psychological problems such as anxiety in response to the viral epidemic and it may decrease their quality of life (QoL). The aim of this study was to explore the influence of healthcare workers' stress and anxiety response to the viral epidemic and their organizational commitment on their QoL. We also explored the mediating effect of resilience on any association.

**Methods:** From January 28, 2021, to January 29, 2021, 389 workers responded to an online survey that included the rating scales Stress and Anxiety to Viral Epidemics-9 items (SAVE-9), Perceived Stress Scale (PSS), Brief Resilience Scale (BRS), Organizational Commitment Questionnaire (OCQ), and the WHO-5 well-being index.

**Results:** A better QoL (WHO well-being index top 25%) of healthcare workers during the COVID-19 pandemic era was predicted by low stress and anxiety in response to the viral epidemic [SAVE-9, adjusted odds ratio (aOR) = 0.92, 95% confidence interval (CI) 0.87–0.97], a high level of resilience (BRS, aOR = 1.26, 95%CI 1.15–1.37), and high organizational commitment (OCQ, aOR = 1.04, 95%CI 1.02–1.07). Mediation analysis showed that resilience partially mediated the effects of stress/anxiety in response to the viral epidemic and the organizational commitment on the quality of life.

**Conclusion:** We observed that the stress and anxiety of healthcare workers in response to the viral epidemic and organizational commitment influenced their QOL. Their resilience mediated the relationship between their psychological problems and QOL.

## Introduction

From December 2019, since the first report of coronavirus disease 2019 (COVID-19) (1), the virus spread rapidly and widely, resulting in a declaration of a global pandemic by the World Health Organization^1^. In South Korea, outbreaks are still occurring in clusters. As of June 24, 2021, there had been 153,155 confirmed cases of COVID-19 in Korea ^2^.

According to previous reports, healthcare workers are likely to experience marked psychological distress during viral epidemics for various reasons. These include their own risk of exposure to the infectious disease, concern about infecting their family members (2–4), and unprecedented and unusual clinical roles imposed on them during the pandemic, which resulted in increased workload burden, reluctance to work, perceived stigmatization, the need to avoid crowds and colleagues, and the feeling of being under surveillance (4–6). Moreover, studies conducted during previous pandemics, such as SARS, influenza A/H1N1, and MERS, have shown that healthcare workers exposed to these conditions are prone to psychiatric comorbidities, such as anxiety, depression, insomnia (3, 4, 7–9), acute stress disorder, or posttraumatic stress disorder (6, 10).

### Stress, Anxiety, and Quality of Life

Psychological distress of healthcare workers also exists in the COVID-19 era. Previous studies reported that a high proportion of healthcare workers are experiencing significant levels of anxiety, depression, insomnia, and distress during the COVID-19 pandemic (11–15). The rates were broadly reported to range between 23.2 and 67.55% for anxiety, 22.8–55.89% for depression, and 34.0%-68.7% for insomnia. The use of different scales and cut-off scores in each study possibly resulted in differences in the reported values. Among healthcare workers, nurses, women, and frontline staff who worked in direct diagnosis and treatment of patients with COVID-19 reported more severe symptoms than others (12). In South Korea, a cross-sectional study of university hospital healthcare workers conducted in the first massive surge in COVID-19 infections showed that direct treatment-related contact with confirmed patients increased the risk of depression and anxiety, while dealing with diagnostic test specimens was also associated with a higher risk of depression (16). It is important to understand that the majority of the healthcare workers experience a wide range of psychological symptoms and their QoL is threatened during the response to viral pandemics.

### Organizational Commitment and Quality of Life

Healthcare worker's organizational commitment also can be associated with their QoL. Organizational commitment can be defined as a state in which people identify with a certain organization, combined with their willingness and desire to remain a member of this organization in order to facilitate organizational objectives (17). Employees' attitude toward their workplace is very important for the development of a particular organization. It has been reported that employee's continuous commitment is associated with their performance and it can lead to better behavioral outcomes, such as work performance and reduced work stress (18). Also, employees' work-related stress affects their organizational commitment, which in turn, affects their performance and productivity (19, 20). Healthcare workers are also employees and their performance is directly related to patients' health status and safety. So, hospitals should attempt to elicit healthcare workers' organizational commitment and enhance their performance by attempting to incorporate the latest technology and up-to-date equipment and to provide adequate staff resources for healthcare workers to prevent fatigue or burnout (21). Consequently, it is important to identify modifiable psychologic distress affecting employee's commitment toward COVID-19 practice in this pandemic.

### Mediating Role of Resilience

Resilience is defined as the ability to adapt to changes in spite of stressful events in a flexible way and to recover from negative emotional experiences (22). Previous studies have reported that a lack of resilience is associated with poor mental health and the pathophysiology of psychiatric symptoms, such as depression and anxiety (23), and it has importance as a predictor of QoL (24). The COVID-19 pandemic is a kind of infectious disease disaster, and resilience is an important dynamic capacity for healthcare workers to maintain their mental health and QoL, and it is necessary for healthcare workers even during normal times (25–27).

There is some evidence of the mediating effects of resilience in relationships between factors regarding healthcare worker's psychological stress during the COVID-19 pandemic. Previous studies of healthcare workers during the COVID-19 pandemic showed psychological resilience has a protective mediating role between depression, perceived stress and personal burnout, and mental health, which may affect the quality of life (28, 29). Similarly, front-line nurses during the COVID-19 pandemic experienced a medium to high level of fatigue but their psychological resilience reduced the negative impact of fatigue on their job satisfaction and turnover intention (30). Nearly half of healthcare providers in HIV clinics report experiencing significant psychological distress, but psychological resilience and institutional support have protective mediating effects between COVID-related stressors and psychological distress, which could disrupt one's quality of life (31). Fear of COVID-19 among healthcare professionals has a negative influence on psychological adjustment skills but resilience limits such influences and plays a protective role against fear of COVID-19 (32).

Distress during the COVID-19 pandemic is inevitable, so it is important to pay attention to the mediating role of resilience for one's well-being. There is a study of student nurses showing a mitigating effect of their psychological resilience that reduced the negative effects of the pandemic-associated stress on their life satisfaction and psychological well-being during the COVID-19 pandemic (33). Also, there is a study reporting the mediating effect of resilience between subjective stress and quality of life in non-healthcare workers not experiencing pandemic circumstances (34). However, to our best knowledge, there is no mediation analysis study evaluating the mediating effect of resilience that focused on quality of life as an outcome for healthcare workers during the COVID-19 pandemic.

In this study, we aimed to explore the influence of healthcare workers' psychological factors such as stress, burnout, anxiety, sleep problems, and their organizational commitment on their QOL. In addition, we explored whether their resilience can mediate the associations among these factors.

## Methods

### Participants and Procedure

This non-contact online survey study was conducted in the ASAN Medical Center (AMC), the largest hospital in South Korea, where a total of 9,216 workers, including 1,759 medical doctors, 4,526 nursing professionals, and 2,931 other workers (health associate professionals, health management and support personnel, clerical support workers, service and sales workers, trade workers, plant or machine operators) are working. The AMC is a tertiary hospital and there are many patients with a high severity of illness compared to other hospitals. Since the confirmed cases inside of the hospital on March 26 and 31, 2020, four wards were in cohort isolation and 57 healthcare workers were quarantined (35). After that, the hospital tried preventing the spread of the virus, but sporadic infection occurred until the end of 2020. This survey was conducted in this situation. Participants were recruited via an advertisement posted on the hospital's intranet, which stated the study's objective, enrollment procedure, and reward for participation. The online survey was completed anonymously and was designed to measure the healthcare workers' stress, anxiety related to the viral epidemic, and their QOL.

From January 28 to 29, 2021, 389 workers voluntarily completed the survey and were given a reward coupon valued about 3 US dollars. The study protocol was approved by the Institutional Review Board (IRB approval number: 2021-0124) of the ASAN Medical Center, and the need for written informed consent was waived. This research complied with the tenets of the Declaration of Helsinki.

### Symptom Assessment

#### Stress and Anxiety to Viral Epidemics-9 Items

The Stress and Anxiety to Viral Epidemics-9 items (SAVE-9) scale was developed by Chung et al.^3^ for the assessment of work-related stress and anxiety responses of healthcare workers in the COVID-19 pandemic era. It has been translated into 15 foreign languages and is currently undergoing validation studies in numerous countries (www.save-viralepidemic.net). The scale includes nine items, which were observed to cluster into two factors: anxiety about the viral epidemic, and work-related stress associated with the viral epidemic. Respondents answered each item on a 5-point Likert scale, ranging from 0 (never) to 4 (always). The appropriate cut-off score of the SAVE-9 scale and anxiety response subscale are 22 and 15 points, respectively^3^.

#### Brief Resilience Scale

The 6-items Brief Resilience Scale (BRS) (36) was developed to measure a person's resilience, i.e., their capacity to recover quickly from difficulties. In this study, the Korean version of the BRS was used (37). Respondents answered each question on a Likert scale, ranging from 1 to 5, with a total score of 6–30.

#### Perceived Stress Scale

The Perceived Stress Scale (PSS) is a self-reporting scale that evaluates the perceived intensity of stress for situations encountered in one's life in the most recent 1-month period (38). The reliability and validity of the scale has been verified in South Korea (39) and the Korean version of the PSS-10 was used in the present study. Each item was assessed on a 5-point Likert scale, ranging from 0 (never) to 4 (very often). The total scores of the scale ranged from 0 to 40, with a higher score indicating a higher degree of subjective stress.

#### Organizational Commitment Questionnaire

The Organizational Commitment Questionnaire (OCQ) assesses the degree of an employee's commitment to the organization. It is a 15-item, self-reported questionnaire developed by Mowday and colleagues (40). This scale assesses 3 factors: (1) a strong belief in, and acceptance of, the organization's goals and values; (2) a readiness to exert effort in serving the organization; and (3) a strong desire to remain with the current organization. Each item is scored on a 7-point Likert scale, ranging from “strongly disagree” to “strongly agree.” A higher score reflects that employees have a greater commitment to the organization.

#### World Health Organization-5 Item Well-Being Index

The World Health Organization-5 Item Well-Being Index (WHO-5) is a 5-item rating scale globally used for measuring subjective psychological well-being and QoL. The WHO-5 is based on the 28-item and WHO-10. Respondents score items as related to the previous 2-week period, from 0 (none of the time) to 5 (all of the time). The final score is calculated by multiplying the raw total score (0–25) by 4 to obtain a score ranging from 0 to 100 (41). Higher scores reflect greater psychological well-being.

### Statistical Analysis

We used SPSS version 21.0 for Windows (IBM Corp., Armonk, NY) for statistical analysis. The clinical characteristics were summarized as mean ± standard deviation. The level of significance for all analyses was defined as 2-tailed *p* < 0.05. To explore factors related to healthcare workers' QoL, participants were categorized into two groups based on their WHO-5 scores: highest quartile of the WHO-5 score (top 25% of participants) and lower three quartiles of the WHO-5 score (bottom 75% of participants). Chi-square tests were used to examine the differences in sex, marital status, shift-work, healthcare job, and COVID-19-related questions, based on the high/low QoL grouping. Student's *t*-test was used to examine differences in age, years of employment, healthcare workers' level of organizational commitment, stress and anxiety to a viral epidemic, perceived stress, and resilience between the high/low QoL groups. Pearson's correlation analysis was used to explore the correlations among age, years of employment, scores of the SAVE-9, PSS, BRS, OCQ, and WHO-5. Logistic regression analysis was used to reveal the variables predicting high organizational commitment or QoL. Finally, the bootstrap method with 2,000 resamples was implemented to explore the mediating effect of resilience on the relationship of psychological status and QoL of healthcare workers.

## Results

The 389 participating healthcare workers included 55 (3.1% of all medical doctors) doctors, 247 (5.4% of all nursing professionals) nursing professionals, and 87 (2.9% of all other workers) other healthcare workers (Table 1). Of the respondents, 335 (86%) were female, 181 (46.5%) were single, and 112 (28.9%) were shift-workers. Their mean age was 35.3 ± 8.0 years, and their mean employment duration was 10.6 ± 8.3 years. Among the subjects, 74 (19.0%) workers had experienced taking care of confirmed COVID-19 patients; only 1 had experienced being infected, although 36 (9.3%) had been quarantined. Additionally, 50 (13.0%) reported that they had experienced or had been treated for depression, anxiety, or insomnia, and 49 (12.6%) considered themselves to be depressed or anxious, or requiring help for their mood state at present.

**Table 1:** Demographic characteristics of the participants (*N* = 389).

	**Mean ± SD, N (%)**
**Healthcare workers**	
Medical doctors	55 (14.1%)
Nursing professionals	247 (63.5%)
Other healthcare workers	87 (22.4%)
**Sex (female)**	335 (86%)
**Age (years)**	35.3 ± 8.0
**Employment duration**	10.6 ± 8.3
**Marital status**	
Single	181 (46.5%)
Married, with children	41 (10.5%)
Married, without children	167 (42.9%)
**Are you a shift-worker? (Yes)**	112 (28.9%)
**COVID-19 questions**	
Have you experienced taking care of confirmed COVID-19 patients? (Yes)	74 (19.0%)
Have you experienced being quarantined due to possible infection with COVID-19? (Yes)	36 (9.3%)
Have you experienced being infected with COVID-19? (Yes)	1 (0.3%)
**Psychiatric history**	
Have you experienced or been treated for depression, anxiety, or insomnia? (Yes)	50 (13.0%)
At present, do you consider yourself to be depressed or anxious, or do you need help for your mood state? (Yes)	49 (12.6%)
**Rating scales**	
Stress and Anxiety to Viral Epidemics-9 items (SAVE-9)	23.0 ± 5.5
SAVE-9 anxiety subscale	15.9 ± 4.1
SAVE-9 work-related stress subscale	7.1 ± 2.2
Perceived stress scale	18.4 ± 3.3
WHO-5 well-being index	45.8 ± 21.4
Brief resilience scale	19.1 ± 3.6
Organizational commitment questionnaire	69.7 ± 13.1

The SAVE-9 total score was 23.0 ± 5.5, and the anxiety subscale and work-related stress subscale scores were 15.9 ± 4.1 and 7.1 ± 2.2, respectively. The PSS and WHO-5 scores were 18.4 ± 3.3 and 45.8 ± 21.4, and the BRS and OCQ scores were 19.1 ± 3.6 and 69.7 ± 13.1, respectively.

The correlations among age, employment duration, and rating scale scores are shown in Table 2. Old age was significantly correlated with long employment duration (*r* = 0.91, *p* < 0.01), high BRS score (*r* = 0.13, *p* < 0.05) and high OCQ score (*r* = 0.29, *p* < 0.01). Long employment duration was significantly correlated with a high OCQ score (*r* = 0.25, *p* < 0.01). A high SAVE-9 scale score was significantly correlated with a high PSS score (*r* = 0.25, *p* < 0.01), low WHO-5 score (*r* = −0.25, *p* < 0.01), low BRS score (*r* = −0.20, *p* < 0.01), and low OCQ score (*r* = −0.12, *p* < 0.05). A high PSS score was correlated with a low OCQ score (*r* = −0.11, *p* < 0.05). A high WHO-5 score was significantly correlated with a high BRS score (*r* = 0.41, *p* < 0.01) and a high OCQ score (*r* = 0.24, *p* < 0.01). A high PSS score was significantly correlated with a low OCQ score (*r* = −0.11, *p* < 0.05).

**Table 2:** Pearson's correlation coefficients for each variable in all subjects (*N* = 389).

**Variables**	**Age**	**Duration of employment**	**SAVE-9**	**PSS**	**WHO-5**	**BRS**	**OCQ**
Age	1.00						
Duration of employment	0.91^**^	1.00					
SAVE-9	−0.02	0.03	1.00				
PSS	0.01	−0.005	0.25^**^	1.00			
WHO-5	0.09	0.04	−0.25^**^	−0.02	1.00		
BRS	0.13^*^	0.04	−0.20^**^	−0.07	0.41^**^	1.00	
OCQ	0.29^**^	0.25^**^	−0.12^*^	−0.11^*^	0.24^**^	0.20^**^	1.00

*SAVE-9, Stress and Anxiety to Viral Epidemics−9 items; PSS, Perceived Stress Scale; WHO-5, World Health Organization-5 well-being index; BRS, Brief Resilience Scale; OCQ, Organizational Commitment Questionnaire*.

** *p < 0.01*.

* *p < 0.05*.

Comparative analysis of the demographic and rating scales scores between subjects with high (top 25%) or low (bottom 75%) QoL are shown in Table 3. Between the two groups, there was no significant difference in age, sex, years of employment, marital status, shift-work, experience of taking care of infected patients, being quarantined, or past psychiatric history. The proportion of workers who currently need help psychologically (*p* < 0.01) was higher in the low QoL group. Workers in the high QoL group reported a lower SAVE-9 score, a higher BRS score, and a higher OCQ score.

**Table 3:** Comparison of demographic variables and rating scale scores between the low and high QOL groups (*N* = 389).

**Variables**	**WHO-5 top-25%** **(***n*** = 89)**	**WHO-5 bottom-75%** **(***n*** = 300)**	***P*** **-value**
	***N*** **(%)**	***N*** **(%)**	
**Healthcare workers (nursing professionals)**	50 (56.8%)	195 (65.4%)	0.166
**Sex (female)**	73 (82.0%)	262 (87.3%)	0.137
**Age (years)**	36.4 ± 8.2	35.0 ± 7.9	0.131
**Duration of employment (years)**	10.9 ± 8.8	10.4 ± 8.1	0.640
**Marital status (single)**	36 (40.4%)	145 (48.3%)	0.117
**Are you a shift-worker? (yes)**	24 (27.3%)	88 (29.4%)	0.402
**COVID-19 questions**			
Have you experienced taking care of confirmed COVID-19 patients? (Yes)	14 (15.7%)	60 (20.0%)	0.230
Have you experienced being quarantined due to possible infection with COVID-19? (Yes)	7 (7.9%)	29 (9.7%)	0.391
Have you experienced or been treated depression, anxiety, or insomnia? (Yes)	8 (9.0%)	42 (14.1%)	0.131
At present, do you consider yourself to be depressed or anxious, or do you need help for your mood state? (Yes)	2 (2.2%)	47 (15.7%)	<0.001
**Rating scales**			
Stress and Anxiety to Viral Epidemics-9 items (SAVE-9)	20.6 ± 5.1	23.7 ± 5.4	<0.001
SAVE-9 anxiety subscale	14.4 ± 4.0	16.4 ± 4.0	<0.001
SAVE-9 work-related stress subscale	6.2 ± 2.2	7.4 ± 2.2	<0.001
Perceived stress scale	18.3 ± 3.1	18.5 ± 3.4	0.615
Brief resilience scale	21.4 ± 3.5	18.4 ± 3.3	<0.001
Organizational commitment questionnaire	75.4 ± 12.3	68.0 ± 12.8	<0.001

Among the subjects, logistic regression analysis revealed that better QoL (WHO well-being index top 25%) among healthcare workers during the COVID-19 pandemic era was predicted by a lack of current psychiatric symptoms [adjusted odds ratio (aOR) = 0.19, 95% confidence interval (CI) 0.04–0.75], low stress and anxiety during the viral epidemic [SAVE-9, adjusted odds ratio (aOR) = 0.92, 95% confidence interval (CI) 0.87–0.97], a high level of resilience (BRS, aOR = 1.26, 95%CI 1.15–1.37), and high organizational commitment (OCQ, aOR = 1.04, 95%CI 1.02–1.07) (Table 4).

**Table 4:** Logistic regression analysis for predicting High QOL (WHO-5, top 25%) in healthcare workers during the COVID-19 pandemic era.

**Variables**	**cOR**	**(95%CI)**	***p***	**aOR**	**(95%CI)**	***P***
At present, do you consider yourself to be depressed or anxious, or do you need help for your mood state? (Yes)	0.13	(0.03–0.54)	0.005	0.19	(0.04–0.75)	0.019
**Rating scale scores**						
SAVE-9	0.90	(0.86–0.95)	<0.001	0.92	(0.87–0.97)	0.001
PSS	0.98	(0.91–1.05)	0.607	1.07	(0.98–1.17)	0.116
BRS	1.31	(1.20–1.42)	<0.001	1.26	(1.15–1.37)	<0.001
OCQ	1.05	(1.03–1.07)	<0.001	1.04	(1.02–1.07)	0.001

*cOR, Crude odds ratio; aOR, adjusted odds ratio, CI, confidence interval; SAVE-9, Stress and Anxiety to Viral Epidemics−9 items; PSS, Perceived Stress Scale, BRS, Brief Resilience Scale; OCQ, Organizational Commitment Questionnaire*.

**Figure 1:**
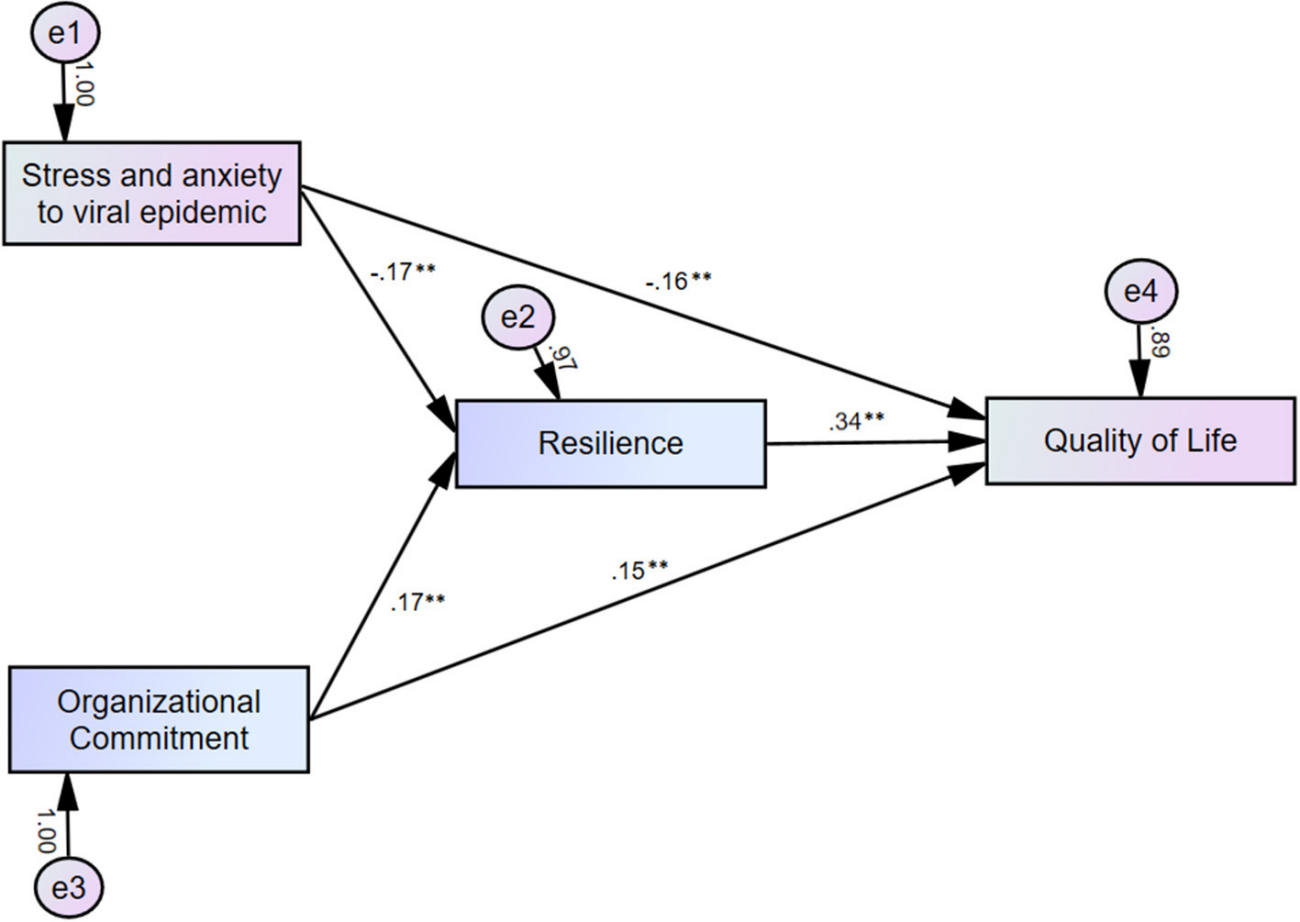
Mediation model showing that the effect of organizational commitment and stress/anxiety in response to the viral epidemic (independent variable) on the quality of life (outcome) is mediated by resilience (mediator). **p < 0.01.

Mediation analysis showed that the complete pathway from organizational commitment and stress/anxiety to viral epidemic (independent variable) to resilience (mediator) to quality of life of healthcare workers (dependent variable) was significant (Table 5). This indicates that resilience partially mediates the effects of organizational commitment and stress/anxiety from the viral epidemic on the quality of life (Figure 1).

**Table 5:** The results of direct, indirect, and total effects on mediation analysis.

**Effect**	**Standardized** **estimator**	**S.E**.	***Z*** **-value**	***p***	**95% CI**
**Direct effect**					
SAVE-9 → QOL	−0.16	0.05	−3.39	<0.001	(−0.25 ~−0.07)
OCQ → QOL	0.15	0.05	3.35	<0.001	(0.06 ~ 0.24)
**Indirect effect**					
SAVE-9 → BRS → QOL	−0.06	0.02	−3.33	<0.001	(−0.10 ~−0.03)
OCQ → BRS → QOL	0.06	0.02	3.28	0.001	(0.03 ~ 0.10)
**Total effect**					
SAVE-9 → QOL	−0.22	0.04	−4.54	<0.001	(−0.31 ~−0.13)
OCQ → QOL	0.22	0.04	4.48	<0.001	(0.12 ~ 0.31)

*QOL, quality of life; SAVE-9, Stress and Anxiety to Viral Epidemics - 9 items; BRS, Brief Resilience Scale; OCQ, Organizational Commitment Questionnaire*.

## Discussion

In this study, we observed that healthcare workers' QoL was influenced by low stress and anxiety about the viral epidemic, high level of resilience, and high organizational commitment. Also, we found that their resilience mediated the influence of the psychological status of healthcare workers on their QoL.

### Work-Related Stress and Anxiety and Organizational Commitment of Healthcare Workers in the COVID-19 Pandemic

The healthcare workers are among the most important people during major public health issues, they have made great contributions to epidemic prevention work and have experienced negative psychological adjustment outcomes, which may increase their baseline level of psychopathology (42), and even decrease their QoL. In particular, it was shown that their level of worry about the epidemic is related to their level of mental health (43). Therefore, early detection, screening, and interventions are crucial. The SAVE-9 is a virus-related anxiety and work stress scale developed by Chung (44) to directly reflect the psychological stress in relation to the viral epidemic during COVID-19. Traditional assessment scales such as the Generalized Anxiety Disorder-seven items (GAD-7) (45), Zung's Self-rating Anxiety Scale (SAS) (46), and the Depression Anxiety and Stress Scale (47) are general scales to evaluate the anxiety of an individual. Although new assessment scales (48–50) have been developed since the COVID-19 pandemic, they are not specific to healthcare workers.

In this study, the mean score of SAVE-9 was 23.0. Considering that our previous study describing the SAVE-9 proposed a cut-off score of 22 points (44), we observed that 241 (62%) of healthcare workers showed work-related stress and anxiety during the pandemic. This finding is consistent with previous studies confirming a substantial proportion of mental health issues among healthcare workers (12, 13, 16, 51). However, due to their low severity of stress and anxiety, the healthcare workers may not be aware of its impact on their QoL. Therefore, concerted efforts to address the psychological care of healthcare workers are essential.

Organizational commitment is a state in which people identify with a certain organization, while having willingness and desire to remain a member of that organization in order to facilitate the organizational objectives (17). In the healthcare system, the organizational commitment of healthcare workers influences their caring behavior, and it has an impact on the patients' health (52). In this study, healthcare workers who were older, had a lower level of burnout, and had a higher QoL showed more organizational commitment. This result is in line with those of previous studies reporting that age and organizational commitment were positively correlated (53–55). However, it needs to be explored further whether age itself is an important factor for organizational commitment, since age is usually significantly related to educational level, years of employment, and promotions. There have also been a number of reports that burnout would reduce commitment to the organization.

Leiter and Maslach (56) investigated the relationship between burnout and commitment of nurses in private hospitals in the US, and found that all dimensions of burnout were significantly correlated with commitment to the organization, and that burnout resulted in less commitment. Jung and Kim (57) also conducted research on the correlation of burnout with organizational commitment and turnover intention of employees in South Korean newspaper companies, and found that individuals with burnout had decreased organizational commitment and increased turnover intention. In the COVID-19 pandemic, it is inevitable that healthcare workers will experience a certain degree of burnout, but as in other reports, those who had less burnout showed better commitment to the organization. Lastly, a significant correlation between organizational commitment and various forms of QoL has been reported in previous studies. Anat et al. (58) found a significant relationship between organizational commitment and QoL at work in public health nurses in Israel. Furthermore, a study conducted among nurses working in prisons showed that work-related QoL affects their organizational commitment and that 20% of the total variance could be explained by work-related QoL (59). Thus, in this viral pandemic era, it is necessary to focus on the burnout level and QoL of healthcare workers to enhance their commitment to their organizations, which is crucial for managing the current crises.

### Mediating Effect of Resilience on the Influence of Stress and Anxiety Response to the Viral Epidemic, and Organizational Commitment on Healthcare Workers' QoL

A better QoL in healthcare workers was predicted by a lower level of anxiety response to the viral epidemic, a lack of current psychiatric symptoms, a higher level of resilience, and higher organizational commitment in the present study. Thus, regulating anxiety or depression in response to the viral epidemic is important to increase the QoL of healthcare workers in this pandemic era. Also, the results of this study showed the possibility of mediating the effect of resilience on the QoL of healthcare workers. Resilience is the capacity for dynamic and successful adaptation to stress and adversity while maintaining normal psychological and physical functioning (60). Resilience plays an important role in remaining mentally healthy and practicing behaviors that can help in coping with anxiety and depression (61). In this study, better resilience among healthcare workers predicted a higher QoL, in accordance with previous studies (24, 62, 63). Finally, the workers' QoL showed a significant association with organizational commitment. Similar results in a previous healthcare worker study showed that their QoL is related to job satisfaction, which is related to organizational commitment (58).

This study was limited in that it was conducted via an anonymous online survey. We used an online survey rather than a face-to-face interview to prevent the possible risk of spreading SARS-CoV-2. Second, more than half of the subjects (63.5%) were nursing professionals, and thus the results do not necessarily reflect the situation of other healthcare workers. Second, only a small proportion of workers (3.1% of medical doctors, 5.4% of nursing professionals, and 2.9% of all other workers) responded to this survey. These are small proportions, which can increase the risk of a type II error. However, the proportions of participants in each group were comparable. Third, we could not obtain any information about the work unit of healthcare workers. It might be considered that the workplace could influence the stress and anxiety response of workers. Especially, a perception of the workplace as being dangerous has been reported to be significantly associated with stress and anxiety in response to the viral epidemic (64). There might be differences between nurses on the frontlines and not on the frontlines. Furthermore, only one participant had experienced being infected, and thus our findings may not reflect the numerous workers who have experienced being infected.

In conclusion, in this study, we observed that the stress and anxiety of healthcare workers in response to the viral epidemic and their organizational commitment influenced their QOL. Also, their resilience mediated the relationship between their psychological problem and QOL. In this COVID-19 pandemic, healthcare workers are suffering from psychological distress, especially work-related stress and anxiety response to the viral epidemic. It is necessary to closely observe their psychological status and develop a psychological support system.

## Data Availability Statement

The raw data supporting the conclusions of this article will be made available by the authors, without undue reservation.

## Ethics Statement

The study protocol was approved by the Institutional Review Board (IRB approval number: 2021-0124) of the ASAN Medical Center. Written informed consent for participation was not required for this study in accordance with the national legislation and the institutional requirements.

## Author Contributions

SC and MA: conceptualization and project administration. HS, KK, and I-KC: data curation. SC, JC, and KHK: formal analysis. HS, JK, and SC: investigations. SC and YH: methodology. KHK and JK: resources. KK, I-KC, and JC: visualization. HS: writing—original draft. KK, I-KC, SC, YH, and MA: writing—review and editing. All authors contributed to the article and approved the submitted version.

## Conflict of Interest

The authors declare that the research was conducted in the absence of any commercial or financial relationships that could be construed as a potential conflict of interest.

## Publisher's Note

All claims expressed in this article are solely those of the authors and do not necessarily represent those of their affiliated organizations, or those of the publisher, the editors and the reviewers. Any product that may be evaluated in this article, or claim that may be made by its manufacturer, is not guaranteed or endorsed by the publisher.

## Footnotes

^1^Available online at: https://www.who.int/emergencies/diseases/novel-coronavirus-2019 (accessed April 20, 2021).

^2^Available online at: http://ncov.mohw.go.kr/en/ (accessed April 20, 2021).

^3^ www.save-viralepidemic.net
